# Kinase Inhibitors and Ovarian Cancer

**DOI:** 10.3390/cancers11091357

**Published:** 2019-09-12

**Authors:** Periklis Katopodis, Dimple Chudasama, Gurleen Wander, Louise Sales, Juhi Kumar, Manreen Pandhal, Vladimir Anikin, Jayanta Chatterjee, Marcia Hall, Emmanouil Karteris

**Affiliations:** 1 Biosciences, College of Health and Life Sciences, Brunel University London, Uxbridge UB8 3PH, UK; periklis.katopodis@brunel.ac.uk (P.K.); dimpz22@hotmail.com (D.C.); 1507909@brunel.ac.uk (L.S.); juhi.kumar@brunel.ac.uk (J.K.); 1306204@alumni.brunel.ac.uk (M.P.); marcia.hall@nhs.net (M.H.); 2 Division of Thoracic Surgery, The Royal Brompton & Harefield NHS Foundation Trust, Harefield Hospital, London UB9 6JH, UK; v.anikin@rbht.nhs.uk; 3 Chelsea and Westminster Hospital NHS Trust, London UB9 6JH, UK; gurleenwander@gmail.com; 4 Department of Oncology and Reconstructive Surgery, Sechenov First Moscow State Medical University, 119146 Moscow, Russia; 5 Faculty of Health and Medical Sciences, School of Biosciences and Medicine, University of Surrey, Guildford GU2 7XH, UK; jayanta.chatterjee1@nhs.net; 6 Mount Vernon Cancer Centre, Rickmansworth Road, Northwood HA6 2RN, UK

**Keywords:** kinase inhibitors, rapalogs, ovarian cancer, mTOR, TKI

## Abstract

Ovarian cancer is fifth in the rankings of cancer deaths among women, and accounts for more deaths than any other gynecological malignancy. Despite some improvement in overall-(OS) and progression-free survival (PFS) following surgery and first-line chemotherapy, there is a need for development of novel and more effective therapeutic strategies. In this mini review, we provide a summary of the current landscape of the clinical use of tyrosine kinase inhibitors (TKIs) and mechanistic target of rapamycin (mTOR) inhibitors in ovarian cancer. Emerging data from phase I and II trials reveals that a combinatorial treatment that includes TKIs and chemotherapy agents seems promising in terms of PFS despite some adverse effects recorded; whereas the use of mTOR inhibitors seems less effective. There is a need for further research into the inhibition of multiple signaling pathways in ovarian cancer and progression to phase III trials for drugs that seem most promising.

## 1. Introduction

Ovarian cancer (OC) is the fifth most common female cancer [1]. It has complex molecular and genetic changes and is a heterogeneous disease that can be categorized into various histological subtypes: High-grade serous (HGSOC) −80% of OCs, low-grade serous, clear cell carcinoma (OCCC), endometrioid, and mucinous adenocarcinoma. The prognosis and chemo sensitivity depend on the subtype. HGSOC, the most common subtype, is characterized by genomic instability and sensitivity to platinum-based chemotherapy [2].

Epithelial ovarian cancer is staged according to the International Federation of Gynecology and Obstetrics (FIGO) staging system where cancer extension beyond the pelvis becomes stage III. Given that the most common serous ovarian cancers are now thought to arise from the fallopian tube, it is unusual to identify patients with earlier stages of ovarian cancer, and the single cells drop off from the fallopian tube and are easily circulated around the peritoneal cavity by peristalsis. Approximately 70% of patients are diagnosed with stage III/IV disease [3,4]. The first-line treatment includes cytoreductive surgery and combined platinum-based chemotherapy [5,6]. Although data suggest that primary surgery achieves better outcomes for patients, this is often challenging, and many patients are treated with primary chemotherapy and interval surgery. Ovarian cancer is highly responsive (>75%) to initial platinum -based chemotherapy but the vast majority (~85%) will recur and ultimately die of recurrent disease [7].

Given the high relapse rate and poor prognosis of advanced stage epithelial ovarian cancer (EOC), exploration into the biology of EOC has burgeoned and led to the development of a number of targeted molecular and biological therapies, including antiangiogenic agents, poly (ADP-ribose) polymerase (PARP) inhibitors, signaling pathway inhibitors, and immunotherapies [6,8].

Due to the advanced stage at diagnosis and genomic heterogeneity of HG serous OC, molecular profile-specific trials for different sub-types need to be developed. For the future, we need to reveal resistance mechanisms, develop rational combinatorial strategies, and identify predictive biomarkers in case we have to impact mortality. We also need to target various other molecular mechanisms involved and altered in this carcinoma.

**Figure 1:**
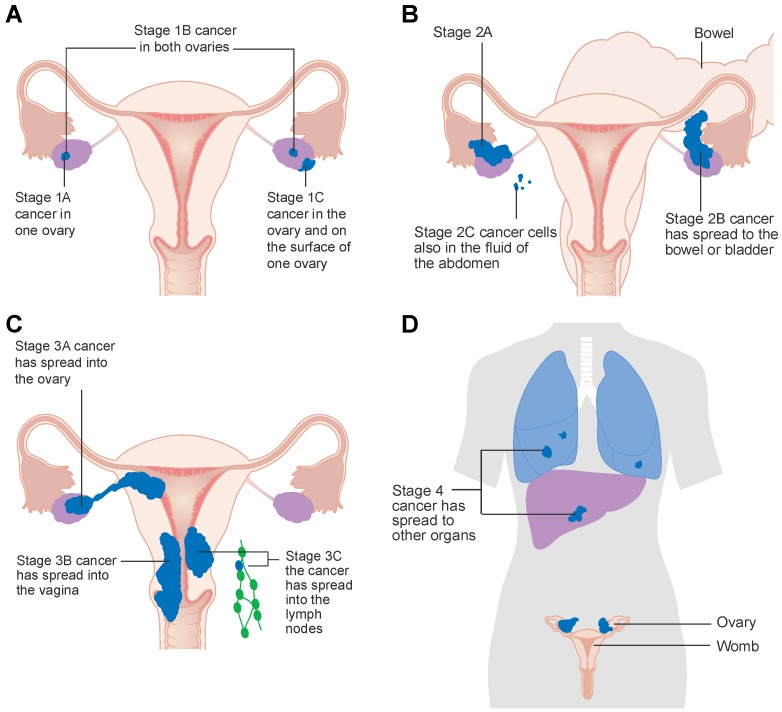
The location and metastasis of ovarian cancer and the corresponding stage. (**A**) Stage I ovarian cancer is confined to the ovaries. (**B**) Stage II ovarian cancer has metastasized to near locations within the pelvic cavity such as the fallopian tubes or bladder, (**C**) stage III ovarian cancer has metastasized to the retroperitoneal lymph nodes or outside of the pelvic cavity, (**D**) stage IV ovarian cancer involves malignant cells in pleural effusion and metastasis to distant sites based on a graphic created by Cancer Research UK.

The severity of ovarian cancer metastasis is currently assessed using the International Federation of Gynecology and Obstetrics (FIGO) staging system detailed below (Table 1; Figure 1).

## 2. Tyrosine Kinases: Current Landscape

Tyrosine-kinases are classified as a group of enzymes that consist of a catalytic subunit, which transfers a phosphate from nucleotide triphosphate to the hydroxyl group of one or more tyrosine residues on signal transduction molecules, resulting in a conformational change affecting protein function. Upon activation, they function to auto-phosphorylate as well as phosphorylate other signaling molecules carrying out an important role in signal transduction and acting to activate and promote a variety of biological processes including cell growth, migration, differentiation, and apoptosis. Amongst the most important cytoplasmic signaling pathways activated are the phosphoinositid 3- kinase/Akt pathway/mechanistic target of rapamycin (PI3K/AKT/mTOR), the Ras/Raf/mitogen-activated protein kinase (MAPK) pathway, the Raf/MEK/ERK1/2 pathway, and the protein kinase C (PKC) pathway. Following the success of a pure vascular endothelial growth factor (VEGF) 1 receptor inhibitor, the monoclonal antibody bevacizumab, it was hoped that the tyrosine kinase inhibitors (TKIs) could target alternate angiogenic pathways in cancer growth. Additionally, the TKIs could potentially be useful in overcoming resistance to VEGF blockade [10].

**Figure 2:**
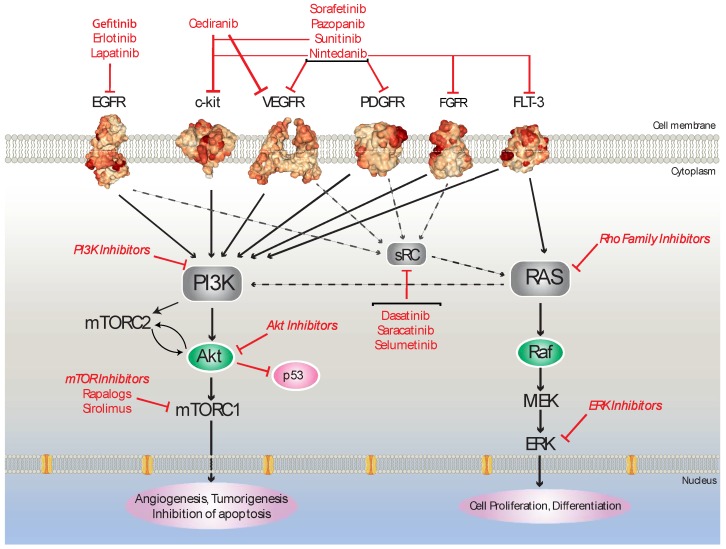
Overview of kinase inhibitors used as potential therapeutic agents against ovarian cancer (OC).

Tyrosine kinase inhibitors (TKIs) utilize different mechanisms such as competing for the substrate and bind in that ATP-binding pocket during an active conformation, they can occupy a site adjacent to the ATP-binding pocket, this allows both the inhibitor and ATP to bind to the same protein and/or bind irreversibly to the protein kinase target [11]. It has also been shown that TKIs can block protein kinase recruitment to the Hsp90-Cdc37 system. This is of particular importance in cancer cells, where these inhibitors deprive oncogenic kinases of access to this complex, leading to their degradation [12] (Figure 2).

## 3. Monotherapy Treatments Using TKIs

**Sorafenib**: Sorafenib is a non-selective multi-kinase inhibitor (Table 2), shown to have anti-proliferative effects in thyroid cancer, renal cell carcinoma, and hepatocellular carcinoma. Sorafenib inhibits signaling in the VEGF and platelet-derived growth factor (PDGF) receptor pathways [13,14,15]. It carries out its function by binding to their substrate and preventing phosphorylation and leading to inhibition of the cell-cycle, consequently attenuating tumor growth. Therefore, sorafenib was also involved in inhibition of the RAS/RAF/MAPK pathway as well as the ERBB (epidermal growth factor receptor; EGFR) signaling pathway through prevention of the coordinated epigenetic switching in these pathways [16,17].

Sorafenib has been shown to have modest anti-proliferative effects in thyroid cancer, renal cell carcinoma and hepatocellular carcinoma [22,23,24,25,26,27,28]. Various adverse side effects have also been reported including diarrhea and edema [20]. It should be noted that despite some modest activity in earlier studies [20], latest findings when used in combination approach are far more encouraging. For example, in a more recent phase II trial, administration of sorafenib plus topotecan in platinum-resistant ovarian cancer demonstrated clinically significant improvement in progression-free survival of these patients [29].

**Sunitinib**: Sunitinib is a highly potent multi-kinase inhibitor. It acts as a competitive inhibitor of the catalytic activity of tyrosine kinase receptors (Table 2) including VEGFRs, PDGFRs, and stem cell factor receptor (c-KIT), to name a few [30]. Phase I and II studies for the clinical efficacy of this TKI for epithelial ovarian cancer have shown acceptable toxicity but only modest activity [30,31].

**Pazopanib**: Pazopanib is an inhibitor of multiple tyrosine kinase receptors and competes with ATP for phosphorylation of the TK receptors. Pazopanib targets various receptors involved in regulating tumor cell growth, metabolism, and angiogenesis, including the VEGF and PDGF receptors (Table 2) [32]. These receptors are integral to the process of angiogenesis, inhibition leads to slower tumor growth, caused by a lack of appropriate blood vessel growth [33].

Pazopanib is not well tolerated in combination with cytotoxic therapy. It has thus only really been studied as a maintenance treatment after chemotherapy. In a study of 940 patients confirmed with cancer of the ovary, fallopian tube, or peritoneum (stages II–IV) were randomized 1:1 to receive pazopanib 800 mg once per day or placebo—after primary chemotherapy—the results were not encouraging since overall survival data did not suggest any extra benefit [34]. Further analyses of data revealed that there were small to no significant mean score differences in global health-related quality of life (HRQoL) between patients receiving pazopanib and placebo [35].

**Nintedanib**: Nintedanib is an oral inhibitor of VEGFRs, PDGFRs, and fibroblast growth factor receptors (FGFRs) (Table 2) [36]. Modest activity has been recorded in early phase II studies of combination or maintenance nintedanib with or after first line chemotherapy [14]. In a recent phase II trial in patients with bevacizumab resistant recurrent EOC, the effect of nintedanib (200 mg/day) was evaluated until disease progression or unacceptable toxicity. The authors of the study concluded that nintedanib as a single-agent has minimal activity in an unselected bevacizumab-resistant EOC population [37].

**Cediranib**: Cediranib is an oral inhibitor of VEGF signaling that binds all three VEGFR and demonstrates selectivity towards VEGFR2. This molecule is a potent ATP inhibitor of VEGF signaling. It does so by binding to the intracellular domains of VEGFRs [38]. Like sunitib, it can also inhibit c-KIT and both PDGFRs (i.e., PDGFRα and PDGFRβ) [38] (Table 2). An initial phase II study of cediranib for recurrent EOC or peritoneal or fallopian tube cancer (dose of 45 mg daily) demonstrated anticancer properties, although this was not very tolerable for patients, with diarrhea being the main concern. Subsequent studies explored the lower doses of 30 and 20 mg daily for five of every seven days. Toxicities similar to other TKIs were also observed including nausea, fatigue, and hypertension [39].

**Tivozanib**: Tivozanib is a pan-VEGFR tyrosine kinase inhibitor that appears to compromise angiogenesis in various tumors [40]. In a recent study, it has been shown that tivozanib reduced cell proliferation in vitro, using chemo resistant EOC cell lines through arrest of G2/M cell cycle and apoptosis [41]. In a phase I study of 41 patients with advanced solid tumors, tivozanib was tolerated well and patients exhibited manageable side effects. This study also shown that this TKI inhibitor was suitable for one dose per day [42]. Tivozanib is currently under investigation in a phase II study in recurrent platinum-resistant ovarian cancer (OC), fallopian tube cancer (FTC) or primary peritoneal cancer (PTC) (TIVO; ClinicalTrials.gov Identifier: NCT01853644). It has been found that the inhibitor is active in patients with recurrent OC, FTC, or PTC, without substantial toxicity, supporting its further development [43].

**Gefitinib**: Gefitinib is an oral aniline quinoline compound which functions through inhibition of EGFR. It has been approved by the FDA for the treatment of non-small cell lung cancer. In vitro, gefitinib has shown to inhibit growth in human ovarian cancer cell lines [44]. In a study on an HPV induced ovarian squamous cell carcinoma where the patient developed bone metastases, the patient did not respond to gefitinib [45]. Results were not that promising for another TKI, named erlotinib (a quinazoline derivative that reversibly inhibits the kinase activity of EGFR). In a randomized phase III study of erlotinib versus observation in patients with no evidence of disease progression after first-line platin-based chemotherapy for ovarian carcinoma, it was shown that maintenance erlotinib after first-line treatment in ovarian cancer did not improve progression-free survival (PFS) or overall survival (OS) [46].

## 4. Combination Trials for TKIs

In a randomized, open-label, phase II trial (MITO 11), PFS was significantly longer in the pazopanib plus paclitaxel group than in the paclitaxel only group (median 6.35 vs. 3.49 months) [33]. Novel role is currently explored in a trial looking at pazopanib and C4AP (fosbretabulin) for ovarian cancer that has come back (PAZOFOS) [46]. In this case there is a combination of an anti-angiogenic agent (AA) with a vascular disrupting agent (VDA). The AA inhibits VEGF signaling that is a major driver of angiogenesis and the VDA is a tubulin-binding agent that affects the cytoskeleton of endothelial cells directly, leading to subsequent disruption of junctions between endothelial cells [47]. A recent a multicenter, multinational, randomized, double-blind, 2-arm, parallel-group, a phase II/III (NCT02641639; FOCUS), evaluating the combination of CA4P plus bevacizumab and chemotherapy in platinum-resistant OC terminated the recruitment status since interim analysis failed to show efficacy benefit.

ICON 6, a placebo-controlled trial of cediranib in combination with second line chemotherapy with or without maintenance cediranib in patients with relapsed OC reported a significant improvement in progression-free survival with minimal alterations in Quality of Life (QoL) [47]. In a more recent randomized phase II study of combination cediranib and olaparib versus olaparib in relapsed platinum-sensitive ovarian cancer, the authors reported that a combination of cediranib (AA) and olaparib (PARP inhibitor) extended PFS significantly, when compared with olaparib alone [48]. Emerging evidence suggests that AAs can drive downregulation of genes implicated in homologous recombination (HR) leading to a creation of a HR-deficient state [48,49,50,51] that can allow a PARP inhibitor to explore further this vulnerability. Olaparib is currently approved as therapeutic agent for BRCA-associated epithelial ovarian cancer, platinum-sensitive recurrence where the BRCA status is unknown, and for germline BRCA (gBRCA) [52]. This is currently being examined further in a larger phase III study ICON 9.

In a randomized, double-blind, placebo-controlled phase 3 trial (AGO-OVAR 12, NCT01015118), the combination of nintedanib with standard carboplatin and paclitaxel chemotherapy was investigated in patients with newly diagnosed advanced ovarian cancer. Median PFS was significantly longer in the nintedanib group (17.2 months) than in the placebo group (16.6 months). However, this subtle increase in PFS was associated with an increased frequency of gastrointestinal adverse events [49]. The authors of the study have proposed that “future studies should focus on improving patient selection and optimisation of tolerability”.

In a phase II, randomized, placebo controlled, multicentre, trial (METRO-BIBF; NCT01610869) the effect of low dose (metronomic) cyclophosphamide with or without nintedanib in relapsed ovarian cancer (ROC) patients was evaluated [50]. As mentioned, angiogenesis has been shown to have a central role in ovarian cancer, both with respect to disease progression and prognosis. The addition of bevacizumab to first-line chemotherapy and as maintenance in certain ovarian cancer patient populations has been shown to improve progression free and overall survival. Several phase II trials of different antiangiogenic drugs have demonstrated activity in patients with relapsed ovarian cancer [53,54]. Oral cyclophosphamide is well tolerated and has been shown to have clinical benefit, since it exhibits anti-angiogenic properties. In this study, addition of nintedanib to cyclophosphamide did not improve OS/PFS. However, in this study of heavily pre-treated ROC, almost one quarter remained on therapy for >6 months, suggesting either more indolent disease and/or cyclophosphamide has longer-term cytostatic or immunological benefits requiring further investigation.

## 5. Concluding Remarks on TKIs

It should be noted that due to the heterogeneous nature of tumors, many treatments are rendered ineffective, whether they are broad-spectrum chemotherapies or more targeted therapies. Despite initial success seen in many targeted therapies [55,56], many of these eventually give rise to resistance and prove ineffective [57,58]. Emerging evidence reveals that targeting multiple pathways may prove more promising [59]. One such includes the RTK inhibitors of the HER family proteins that have been evaluated clinically include the EGFR TKIs gefitinib and erlotinib, including the dual therapies such as EGFR/HER2 TKI lapatinib, and the EGFR/VEGFR/RET TKI vandetanib. Clinical trials have proved disappointing for HER TKIs in EOC patients either as a single agent or in combination with chemotherapy or other biological agents [60,61,62,63,64,65,66,67].

There is growing evidence that inhibiting a single step in one pathway does not necessarily prevent downstream events from occurring, as these could be triggered from another cascade, leading to resistance to the initial therapy. The so-called “horizontal” blockade looks to overcome this, where two or more TKIs or other inhibitors are combined to target multiple pathways [68]. Thus in this context, the “vertical” blockade is also included, whereby several steps of the same pathway could be inhibited, preventing the negative feedback loops that occur in case of a single step inhibition [69]. Some of the more successful reported drugs include nintedanib, pazopanib and cediranib, all multi-kinase inhibitors.

## 6. Inhibitors of Src Kinase

There is also evidence for activation of Src and MAPK in high-grade serous OC (HGSOC) [58]. Src belongs to a family of nine non-receptor tyrosine kinases (Src, Lyn, Fyn, Lck, Hck, Fgr, Blk, Yrk, and Yes), sharing a key role in many cellular signaling pathways [70]. Extensive research on the role of the Src family kinase (SFK) has shown that it can control four key cellular functions, namely: Cell adhesion, proliferation, invasion, as well as cell motility [71,72]. Src can be induced via interaction with activated EGFR [73], HER2 [74], FGFR [75], or hepatocyte growth factors [76] leading to changes in tertiary Src conformation [70]. Interestingly, over-expression and subsequent activation of SFKs has been documented in human ovarian cancer in vitro [77]. When clinical samples were examined, it has been shown that 50% of tumors have some deregulation on the Src signaling pathway that is also associated with poor prognosis [78]. Dasatinib is a tyrosine kinase inhibitor that inhibits the Src family kinases as well as focal adhesion kinases (FAK; [79]) and EphA2 [80] at low concentrations [79].

Konecny et al. showed an anti-proliferation effect of dasatinib across all 34 ovarian cancer cell lines tested, but showed variation of up to three log-fold differences between the cell lines [70]. Dasatinib was shown to significantly inhibit invasion and induce apoptosis in vitro [81]. Src inhibition by Selumetinib rapidly mediates MEK/MAPK activation in preclinical breast cancer models [82,83]. Selumetinib added to saracatinib overcomes the EGFR/HER2/ERBB2–mediated bypass activation of MEK/MAPK that is observed with saracatinib alone and targets tumor-initiating ovarian cancer populations, supporting combined Src–MEK inhibition therapeutics for future trials [58]. However, in a placebo-controlled trial of weekly paclitaxel and saracatinib in platinum-resistant OC in a total of 107 patients, saracatinib did not improve outcomes of weekly paclitaxel in platinum-resistant OC [84].

## 7. Inhibitors of the Mechanistic Target of Rapamycin (mTOR) Pathway

The PI3K/AKT/mTOR is one such cellular signaling pathway implicated in many cellular activities including regulation of cell growth, motility, survival, proliferation, protein synthesis, autophagy, transcription, as well as angiogenesis. It is one of the most investigated intracellular signaling pathways. Consistent with its physiological role, the PI3K/AKT/mTOR pathway has been found to be hyperactivated in many types of cancer. Overall, this pathway is dysregulated via several genetic mechanisms in approximately 30% of solid cancers [85]. It plays a critical role in the malignant transformation of human tumors and their subsequent growth, proliferation, and metastasis. It is frequently activated in OC, especially in clear cell carcinoma and endometrioid adenocarcinoma. As a sign of its dysregulation, PIK3CA mutations have been reported in approximately 12% of OCs. The mTOR pathway is activated in approximately 70%. The type of PI3K alteration seems to be related to the histology. PTEN loss has been identified in 5% of cases of HGSOC and amplifications in PIK3CA in 20% and in one of the AKT isoforms (AKT1, AKT2, and AKT3) in 10–15% of cases [86,87]. A number of genetic aberrations in PI3K/AKT/mTOR signaling genes have been found in EOC, including PTEN, INPP4B, PIK3CA, PIK3R1, AKT1, AKT2, TSC1, TSC2, and mTOR [88]. These observations have directed increasing interest in evaluating inhibitors of this pathway as a form of therapy for EOC. Recent studies including The Cancer Genome Atlas program have provided a more detailed understanding of the roles played by PI3K pathway aberrations in ovarian cancer. In high-grade serous ovarian carcinoma, the mutation of PIK3CA and AKT, or inactivating mutations in the PTEN gene are rare [89].

mTOR is a serine/threonine protein kinase. mTOR, a 289 kDa highly conserved serine/threonine kinase, is the central catalytic component of mTOR Complex 1 (mTORC1) and mTOR Complex 2 (mTORC2). These two complexes have distinct functions and associated proteins [90]. mTORC1 contains mTOR, Raptor, DEPTOR, GBL, and PRAS 40. mTORC2 contains mTOR, Rictor, DEPTOR, and GBL [91].

PI3K/AKT/mTOR inhibitors not only impact directly upon cancer cells but can also affect immune cell effector function and to modulate the tumor microenvironment. As single agent therapies, the efficacy of PI3K/AKT/mTOR inhibitors in the treatment of a variety of cancers has generally not been satisfactory and phase III clinical trials have not been reported yet in patients with ovarian cancer [92,93,94,95,96].

## 8. Monotherapy Trials Using Rapalogs (mTOR Inhibitors)

Rapamycin (also known as sirolimus), the first known inhibitor of mTOR kinase, was first described in 1975 in two seminal papers [97,98]. It was initially developed as an antifungal and immunosuppressive drug, but its anticancer potential was observed during the last decade. Since the discovery of Rapamycin, a host of semi-synthetic rapamycin-related mTOR inhibitors, known as rapalogs, have been developed by modifying the C40 hydroxyl group to improve the aqueous solubility and pharmacokinetics of Rapamycin. These include everolimus, ridaforolimus, and temsirolimus, among others. The mechanisms by which Rapamycin (a first generation mTOR inhibitor) exerts its effects if by binding to FRBP-12 (12 kDa FK506-binding protein) and forming a ternary complex with mTOR, leading to inactivation of mTOR compex 1 (mTORC1) [99].

A few rapalogs (Table 3) are currently used in clinical trials, out of which temsirolimus and everolimus have been granted FDA approval [92,93,94]. However, only modest therapeutic effects have been observed in all these malignancies since mTOR inhibitors may have more of a cytostatic rather than a cytotoxic effect, with a benefit mainly in terms of disease stabilization and progression free survival (PFS) improvement rather than tumor shrinkage. The phase I and II trials conducted to date with mTOR inhibitors in OC have been single-arm studies in which the primary efficacy end point was usually objective response rate (ORR).

The most studied mTOR inhibitors in OC are: Temsirolimus, everolimus, and ridaforolimus. Initial studies have all been phase I and phase II trials with limited number of patients using one of these agents as monotherapy.

In a phase II clinical trial conducted by the Gynecologic Oncology Group, it was shown that temsirolimus exerted modest activity in patients with recurrent epithelial ovarian and primary peritoneal cancers. The progression free survival response was such that did not lead to inclusion for a phase III trial [95].

The most common toxicities were metabolic toxicities, fatigue, and interstitial pneumonitis. A second phase II study of temsirolimus in patients with platinum-refractory/resistant EOC was stopped early for lack of efficacy [96]. In this study led by the AGO-study group in patients with ovarian cancer and endometrial carcinoma, the efficacy of temsirolimus did not actually meet the predefined criteria [96].

Considering the limited activity of mTOR inhibitors as monotherapy and the evidence from preclinical studies indicating an additional benefit of mTOR inhibitors when associated with chemotherapy, some trials have investigated the effects of the combination of mTOR and cytotoxic drugs [100].

## 9. Combination Trials

In a phase I study, 41 patients with advanced gynecologic malignancies were administered a combination of bevacizumab (VEGF inhibitor) plus temsirolimus (mTOR) inhibitor. Using FDA-approved doses, 20% of patients achieved stable lasting over six months [101]. Similarly, in another phase I study, 32 patients with advanced solid tumors were treated with bevacizumab, everolimus, and panitumumab. The doses were tolerable and moderate clinical activity was recorded [102]. Similar results were reported when patients with advanced/or recurrent gynecologic malignancies were treated with temsirolimus and topotecan (a topoisomerase inhibitor). Although there was evidence that 9 out of 11 patients reported stable disease covering approximately a two-year study period, the authors concluded that “the regimen may be safe in women who have not previously received radiation, but full doses of each agent could not be administered in combination” [103]. Despite the limited therapeutic success in ovarian cancer, new rapalogs are now emerging. Despite lack of any phase I studies, there are some promising results using a dual mTORC1/2 inhibitor named WYE-*132* in vitro. Preliminary results show that this compound can stop proliferation of ovarian cancer cells via mTOR-dependent and mTOR-independent signaling pathways [104].

A Phase II clinical trial investigating the effects of the combination “bevacizumab plus everolimus” in recurrent ovarian cancer patients (NCT01031381), revealed that 14/50 (28%) patients were progression-free at six months (95% CI 16.67–42.71%), with 5 (0.65%) grade 4 and 66 (8.64%) grade 3 toxicities, mostly consisting in oral mucositis, fatigue, abdominal pain, diarrhea, nausea, and hypertension [105]. The toxicity profile of mTOR inhibitors for OC patients needs further assessment. Larger studies on breast cancer patients suggest that the most common adverse events of mTOR inhibitors include stomatitis (all grades: Approximately 60%), non-infectious pneumonitis (15%), rash (40%), hyperglycaemia (15%), and immunosuppression (40%) [106,107].

Vistusertib is a dual mTORC1/mTORC2 inhibitor, competitively binding to the ATP site [108]. Two recent studies assessed the combinatorial effect of vistusertib and paclitaxel. A combination of vistusertib and paclitaxel on inhibition of cell growth was additive in a majority of 12 OC cell lines (*n* = 12) studied, followed by reduction of S6 and AKT phosphorylation [109]. In the same study, in a cisplatin-resistant xenograft model, there was a significant reduction in tumor volume only in the group that was treated with both paclitaxel and vistusertib. Results from a phase I trial of vistusertib in combination with paclitaxel in patients (*n* = 22) with GHSOC and squamous non-small-cell lung cancer also appeared to be encouraging. In the OC cohort, RECIST (Response Evaluation Criteria in Solid Tumors) rates were 52% and median PFS was 5.8 months. However, further clinical trials should be explored for knowing the pharmacodynamics and pharmacokinetics of vistusertib [110].

## 10. Future Perspectives on mTOR Inhibition and OC

Studies on the mTOR field over the past 20 years underline a high level of complexity in this particular signaling, its inhibition and expression of key mTOR components in a tissue- and cell-specific manner. Initial studies from our laboratory revealed a differential expression of expression of mTOR signaling components in drug resistance using in vitro OC models. We showed that RICTOR and mTOR expression were up-regulated in the PEO1 taxol-resistant cells (TaxR; cells with epithelial phenotype), whereas their expression was markedly down-regulated in SKOV-3TaxR OC cells (cells with intermediate mesenchymal phenotype) [111]. This is of increasing significance since epithelial–mesenchymal transition (EMT) appears to facilitate the invasive OC phenotype [112].

BEZ (BEZ-235) is another dual inhibitor for PI3K and the mTOR complex, it works by competitively binding to both of their ATP sites [113]. We assessed its effect in vitro using two OC cell lines (SKOV3 and MDAH-2774 cells) [114]. We showed that BEZ reduced cell proliferation, and this is accompanied by dephosphorylation of S6K (Thr^389^). We highlighted then that the need for ‘tailor-made’ therapies against OC depending on the genetic make-up of the patient.

It should be noted that despite a wealth of preclinical/clinical data on PI3K/AKT/mTOR pathway inhibitors in OC, currently there are no FDA approved inhibitor(s) as combinatorial treatments for ovarian cancer Interestingly, the PI3K inhibitors copanlisib and idelalisib (for follicular lymphoma) have been clinically approved [115,116,117]. There is also evidence that protein kinase C (PKC) can activate the mTORC1 signaling pathway [118]. It would have been interesting to test whether dual inhibition of mTOR and PKC pathways can be of benefit to ovarian cancer patients. However, emerging data on the clinical use of PKC inhibitors are not very encouraging. For example, efforts to target PKC signaling in clinical trials for pancreatic cancer have failed [119]. Similarly, in a phase II study for multiple myeloma, enzastaurin (a serine/threonine PKC inhibitor) was not effective in this particular cohort [120]. The last study recorded using the same inhibitor in another phase II study in patients with recurrent epithelial ovarian cancer and primary peritoneal carcinoma was not promising either [120].

For the future, large-scale investigations are needed for a better characterization of their properties as antitumor agents. To date, no phase III trials have been reported on these drugs. Moreover, defining the OC population by the sub-types will tell us which subset shall derive maximal therapeutic benefits with minimal adverse effects.

## 11. Conclusions

Over the past decade we have gained a better insight into the molecular mechanisms implicated in the aetiopathogenesis of ovarian cancer. Looking at the current landscape, combinatorial treatments appear to be more beneficial than single agents for ovarian cancer patients (Figure 2). However, further research is needed not only for phase III trials but there is also a need for the development of biomarkers that will predict response or relapse. Moreover, sequencing of the human genome has allowed the development of new therapeutic regimes; e.g., PARP inhibitors for ovarian cancer patients carrying BRCA mutations. Hopefully, the field will move towards personalized medicine, where tailor-made treatments will become available.

## Figures and Tables

**Table 1:** Ovarian cancer staging parameters as defined by the International Federation of Gynecology and Obstetrics (FIGO) [9].

Stage	Substrate	Description
**I**	**Ia**	The tumor is confined to one ovary with no signs of tumor on the surface
	**Ib**	As Ia but involving both ovaries
	**Ic**	The tumor is confined to one or both ovaries with either or all the following: signs of the tumor on the surface of the ovary, rupture of tumor capsule before or during surgery, malignant cells found in ascites
**II**	**IIa**	Metastasis outside the ovaries in the uterus or fallopian tubes
	**IIb**	Metastasis to pelvic cavity organs for example the bladder
**III**	**IIIa**	Metastasis to retroperitoneal lymph nodes or microscopic malignancy found outside the pelvis
	**IIIb**	Tumor smaller than or equal to 2cm found outside the pelvic cavity including surface of liver and/or spleen
	**IIIc**	Tumor bigger than 2cm found outside the pelvic cavity including surface of liver and/or spleen
**IV**	**IVa**	Pleural effusion (fluid around the lungs) positive for malignant cells
	**IVb**	Metastasis to distant sites including extra-abdominal and parenchymal liver or spleen involvement

**Table 2:** List of tyrosine kinase inhibitors (TKIs) and their targets.

Agent	VEGFR	PDGFR	EGFR	FGFR	C-kit	Flt-3
Sorafenib	**✔**	**✔**				
Sunitinib	**✔**	**✔**			**✔**	
Pazopanib	**✔**	**✔**				
Nintedanib	**✔**	**✔**		**✔**	**✔**	**✔**
Cediranib	**✔**				**✔**	
Tivozanib	**✔**					
Gefitinib			**✔**			
Erlotinib			**✔**			
Lapatinib			**✔**			

EGFR: epidermal growth factor receptor; FGFR: fibroblast growth factor receptor; PDGFR: platelet-derived growth factor receptor; VEGFR: vascular endothelial growth factor receptor; c-kit: mast/stem cell growth factor receptor; Flt-3: FMS-like tyrosine kinase 3 [18,19,20,21].

**Table 3:** Molecular structures, chemical formulas, and uses of rapalogs commonly used.

Agent	Mechanism of Action	Molecular Structure	Chemical Formula	Licenced Uses
**Rapamycin** **(Sirolimus)**	Forms complex with FKBP12 to allosterically inhibit mTOR	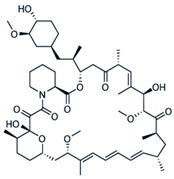	C_51_H_79_NO_13_	Rapamune^®^ (Pfizer) To prevent organ rejection
**Everolimus** **(RAD001)**	mTOR inhibitor Antineoplastic chemotherapy drug	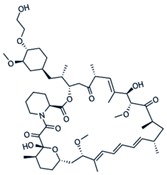	C_53_H_83_NO_14_	Afinitor^®^ (Novartis) Advanced kidney cancer, TSC-associated brain tumors, advanced hormone receptor-positive, HER2- negative breast cancer, neuroendocrine tumours (NET) in pancreas, lung, GI
**Ridaforolimus** **(Deforolimus)**	mTOR inhibitor	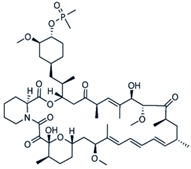	C_53_H_84_NO_14_P	EluNIR^®^ Ridaforolimus Eluting Coronary Stent System (Medinol Ltd.)
**Temsirolimus** **(CCI-779)**	mTOR protein inhibitor, antineoplastic	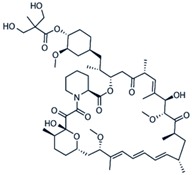	C_56_H_87_NO_16_	Torisel^®^ (Pfizer) Treatment of advanced renal cell carcinoma
